# miR-200c Inhibits invasion, migration and proliferation of bladder cancer cells through down-regulation of BMI-1 and E2F3

**DOI:** 10.1186/s12967-014-0305-z

**Published:** 2014-11-04

**Authors:** Lei Liu, Mingning Qiu, Guobin Tan, Ziji Liang, Yue Qin, Lieqian Chen, Hege Chen, Jianjun Liu

**Affiliations:** Laboratory of Urology, Guangdong Medical College, Zhanjiang, 524001 China

**Keywords:** miR-200c, BMI-1, E2F3, Bladder cancer cells

## Abstract

**Background:**

MicroRNA-200c (miR-200c) is one of the short noncoding RNAs that play crucial roles in tumorigenesis and tumor progression. It also acts as considerable modulator in the process of epithelial-to-mesenchymal transition (EMT), a cell development regulating process that affects tumor development and metastasis. However, the role of miR-200c in bladder cancer cells and its mechanism has not been well studied. The purpose of this study was to determine the potential role of miR-200c in regulating EMT and how it contributed to bladder cancer cells in invasion, migration and proliferation.

**Methods:**

Real-time reverse transcription-PCR was used to identify and validate the differential expression of MiR-200c involved in EMT in 4 bladder cancer cell lines and clinical specimens. A list of potential miR-200 direct targets was identified through the TargetScan database. The precursor of miR-200c was over-expressed in UMUC-3 and T24 cells using a lentivirus construct, respectively. Protein expression and signaling pathway modulation were validated through Western blot analysis and confocal microscopy, whereas BMI-1 and E2F3, direct target of miR-200c, were validated by using the wild-type and mutant 3′-untranslated region BMI-1/E2F3 luciferase reporters.

**Results:**

We demonstrate that MiR-200c is down-regulated in bladder cancer specimens compared with adjacent ones in the same patient. Luciferase assays showed that the direct down-regulation of BMI-1 and E2F3 were miR-200c-dependent because mutations in the two putative miR-200c-binding sites have rescued the inhibitory effect. Over-expression of miR-200c in bladder cancer cells resulted in significantly decreased the capacities of cell invasion, migration and proliferation. miR-200c over-expression resulted in conspicuous down-regulation of BMI-1and E2F3 expression and in a concomitant increase in E-cadherin levels.

**Conclusions:**

miR-200c appears to control the EMT process through BMI-1 in bladder cancer cells, and it inhibits their proliferation through down-regulating E2F3. The targets of miR-200c include BMI-1 and E2F3, which are a novel regulator of EMT and a regulator of proliferation, respectively.

**Electronic supplementary material:**

The online version of this article (doi:10.1186/s12967-014-0305-z) contains supplementary material, which is available to authorized users.

## Background

Bladder cancer is one of the most lethal urological malignant tumors worldwide [1]. A multistep accumulation of genetic and epigenetic factors result in uncontrolled cellular invasion, migration, proliferation, increased cell survival, and metastatic spread. Although the treatment of bladder cancer has improved greatly in recent years, the incidence of this disease is gradually increasing. Recent advances in the use of high-throughput methods of molecular analysis have made it easier to identify genetic profiles characteristic of distinct tumor types and to identify targets and pathways that may underlie a particular clinical behavior; however, systemic chemotherapy remains palliative and only modestly effective [2].

A biological process known as epithelial-to-mesenchymal transition (EMT) has been reported as having a role in tumor invasion, migration and metastasis [3]. EMT is characterized by losing epithelial cell marker such as E-cadherin, and gaining of a mesenchymal phenotype with expression of mesenchymal proteins including vimentin [4]. During EMT, down-regulation of E-cadherin induces epithelial cells undergoing changes in cell morphology and motility so that they adopt mesenchymal characteristics [5]. Expression of E-cadherin is controlled by several transcriptional repressors, including Twist, Snail1, Snail2/Slug, E47, ZEB1/TCF8, and ZEB2/SIP1, which bind to E-boxes in the E-cadherin promoter [6]. The transcriptional repressors also induce the mesenchymal genes N-cadherin and Vimentin [7].

MicroRNAs (miRNAs) are noncoding RNAs of approximately 20–22 nucleotides that function in post-transcriptional gene regulatory pathways [8]. Alterations in miRNA expression have been described in many different human tumors, and numerous studies have demonstrated that miRNAs function as key pathogenic components, impacting cancer cell growth, survival, and the capacity to metastasize [9-14]. Recent studies have shown that ZEB1 and ZEB2 are direct targets of the miR-200 family in human breast cancer cells (MDA-MB-231), canine kidney cells (MDCK), murine models, and a NCI-60 panel of cell lines [15-17]. The down-regulation of ZEB1 and ZEB2 increased E-cadherin expression; in contrast, loss of miR-200, which occurs in many different human cancers, including breast cancer [18], ovarian cancer [19], prostate cancer [20], and endometrial carcinoma [21], results in increased ZEB1/ZEB2 and repression of E-cadherin [16,22-24]. This latter change is coincident with more aggressive biological behavior in cancer (increased invasion and migration) [16,18,24,25]. Hundreds of miRNAs are encoded in the human genome, with an estimated 30% of mRNAs possessing conserved miRNA-binding sites, suggesting that miRNA-based regulation is an integral component of the global gene expression program [26].

The Polycomb group (PcG) of proteins comprises an important class of transcriptional repressors that orchestrate changes in chromatin structure to regulate gene activity, and many of the PcG proteins demonstrate altered expression in human cancers [27,28]. BMI-1 is a PcG protein that has been shown to be an important transcriptional repressor of the Ink4a/Arf gene locus [29-31], which encodes two separate gene products (p16 ink4a and p14 Arf) from two distinct reading frames. P16ink4a inhibits CDK activity and, thereby, blocks entry into the cell cycle by preventing phosphorylation of the retinoblastoma protein (Rb) by cyclin D-CDK4/6 complexes. P14 ARF arrests cell cycle progression and promotes apoptosis by promoting the stability of p53 [32]. BMI-1 also plays a critical role in the maintenance of stem cells [33]. Consistent with these observations suggesting an important oncogenic role for BMI-1, and up-regulated BMI-1 expression has been demonstrated in numerous human cancers [27,28], including bladder cancer [34]. High expression of BMI-1 has been shown to be associated with poor prognosis in bladder cancer [35].

E2F3, a key regulator of G1/S transition, plays major roles in regulating both cell proliferations [36]. More recently, several miRNAs, such as miR-34a, miR-20a and miR-125b have been reported to inhibit cell proliferation by negatively regulating E2F3 in tumor cells [37-39]. Notably, E2F3 is over-expressed in almost all bladder cancer and cell lines [40,41], suggesting that E2F3 is important for human bladder cancer development.

The aim of this study was to investigate the mechanisms underlying the EMT process in bladder cancer. Our results revealed a close, direct association between expression of miR-200c, BMI-1, E-cadherin, N-cadherin, P14, P16 and E2F3 expression, representing a pivotal cellular axis impacting not only the capacity of bladder cancer cells to metastasize but also the ability of proliferation. In this study, we found that down-regulated expression of miR-200c and up-regulation of its direct target BMI-1 and E2F3 in bladder cancer tissue and cell lines. MiR-200c suppressed tumorigenesis through targeting BMI-1 and E2F3.

## Methods

### Cell lines and cell culture

Human bladder cancer cell lines (UMUC-3 and T24) were purchased from Guangzhou Jennio Biological Technology Co., Ltd, Guangzhou, China. All cell lines were maintained in high glucose DMEM medium (GIBCO, Gaithersburg, MD, USA) supplemented with 10% fetal bovine serum (FBS) at 37°C with an atmosphere of 5% CO_2_ in humidified air.

### Tissue specimens

Clinical tissue samples used in this study were histopathologically and clinically diagnosed at the Affiliated Hospital of Guangdong Medical College (Zhanjiang, China) from 2012 to 2014. For the use of these clinical materials for research purposes, prior patients’ consents and approval from the Institutional Research Ethics Committee were obtained.

### RNA Isolation and quantitative RT-PCR

Total RNA and miRNA were isolated using the Total RNA kit (Omega Bio-tek, Inc, Guangzhou, China) and miRNA kit (Omega Bio-tek, Inc, Guangzhou, China) respectively followed by cDNA synthesis with 5 ng of total RNA using the TaqMan miRNA Reverse Transcription Kit (TaKaRa). BMI-1, E2F3, E-cadherin, N-cadherin, Vimentin, p16INK4a, and p14ARF expression levels in cells and clinical specimens were quantified by real-time quantitative PCR performed with SYBR Premix Ex Taq II (TaKaRa; Dalian, Liaoning, China). PCR was carried out with a two-step qRT-PCR with specific primers for GAPDH (as internal control) at 95°C for 30s, followed by 40 cycles of amplification at 95°C for 5 s and 56°C for 30s. All results were representative of three independent assays, and the expression levels of miRNAs and mRNAs were expressed as 2^-ΔΔCT^. The designed specific primers were listed in Table 1.

**Table 1:** **Sequences for target gene primers for qRT-PCR**

**Gene**	**Primer sequence 5′-3′**	**Tm (°C)**
RNU6B	F: CTCGCTTCGGCAGCACA	59.42
	R: AACGCTTCACGAATTTGCGT	55.75
miR-200c	F: TAATACTGCCGGGTAATGATGG	58.21
	R: TCGTATCCAGTGCAGGGTC	59.72
GAPDH	F: TGCACCACCAACTGCTTAG	60.07
	R: AGTAGAGGCAGGGATGATGTTC	59.72
Bmi1	F: TGGATCGGAAAGTAAACAAAGAC	56.60
	R: TGCATCACAGTCATTGCTGCT	58.01
E2F3	F: TGCCTGACTCAATAGAGAGCCTAC	61.97
	R: TCCCATTGTGGTCTTGGTTGT	58.01
E-cadherin	F: GAAAGCGGCTGATACTGACC	59.85
	R: CGTACATGTCAGCCGCTTC	59.72
Vimentin	F: GGGAGAAATTGCAGGAGGAG	59.85
	R: AGGTCAAGACGTGCCAGAGAC	61.92

### Western blot analysis

Western blotting was conducted using standard procedures, using anti-BMI-1 (40kD, 1:20000, Abcom; Cambridge, USA), anti E2F3 (37kD, 1:2000, Abcom; Cambridge, USA), anti-E-cadherin (135kD, 1:1000, Cell Signaling Technology; Boston, USA), anti-N-cadherin (140kD, 1:1000, Abcom; Cambridge, USA), anti-Vimentin, anti-p16INK4a (16kD, 1:500, Abcom; Cambridge, USA), anti-p14ARF (14kD, 1:500, Cell Signaling Technology; Boston, USA), or anti-β-tubulin antibody (55kD, 1:50000, Pure Earth Biotechology Co. Ltd.; Guangzhou, China, as a loading control), respectively. And then the membrane strips were probed with a secondary antibody (1:10000, Pure Earth Biotechology Co. Ltd.), β-tubulin was used as a loading control. Relative protein levels were quantified by scanning densitometry (Alpha View SA 3.4.0, ProteinSimple, USA), and the relative gray value of protein was calculated as band intensity of protein of interest/band intensity of loading control.

### Infection of miRNAs

Vector control (pEZX-MR03, GeneCopoeia; Guangzhou, China), miR-200c over-expression plasmid (pEZX-MR03, GeneCopoeia; Guangzhou, China), and miR-200c inhibit plasmid (CMV- RFP- U6- miRNA inhibitor-PGK-puromycin, GeneCopoeia; Guangzhou, China) were transfected into 293 T cells, and viral supernatants were collected 48 h after transfection to infect human bladder cancer cells (UMUC-3, T24). After a 72-hour infection period, miR-200c-infected cells were seen accounting approximately 70% in the dish at which point we change the culture medium containing puromycin at 1 μg/ml for about 48 h post-transfection.

### Luciferase reporter assay

3′-Untranslated region (3′-UTR) reporter plasmids for miR-200c were constructed via insertion of miR-200c seed sequence into the XbaI restriction site 3′ to luciferase gene in the pGL3-control plasmid (Promega, Madison, WI, USA). Briefly, HEK-293 cells were seeded in 24 well cell culture clusters (Corning Incorporated; Corning, NY, USA). When reached 70% confluences, the cells were co-transfected with hsa-miR-200c/control miRNA and wild/mutated 3’-UTR of BMI-1, E2F3. After 48 h of transfection, the cells were harvested for firefly and Renilla luciferase activity assay. The Renilla luciferase activities were used to normalize the transfection efficiency.

### Cell proliferation assay

Cells (5 × 10^3^) transfected with control vectors, miR-200c over-expression, and miR-200c inhibitor vector were plated in 96-well plates (Corning Incorporated; Corning, NY, USA) for 6 days, separately. The CCK-8 reagent was added into each well of the 96-well assay plate each day and incubated for 2 h. The absorbance was measured at 450 nm using a 96-well plate reader.

### Colony formation assay

Colony formation assay was performed by a slightly modified method, briefly, 1000 cells were seeded into 60 mm plastic dishes (Nest Biotechnology, Hong Kong, China), and cultured with 3 mL DMEM medium supplemented with 10% FBS at 37°C under an atmosphere of 5% CO_2_ in humidified air (UMUC-3 cells were cultured for 7 days and T24 cells were cultured for 12 days, respectively). The numbers of colonies were counted after staining with Coomassie Brilliant Blue. All studies were conducted with 3 replications.

### *In vitro* wound healing (migration) assay

UMUC-3 and T24 cells (5 × 10^5^) were plated in 6-well plates and cultured until they reached confluence. A diametric scratch was done using a pipette tip followed by two culture medium changes. Cells were photographed in several pre-marked spots as 0 h. Multiple photographs were then taken at 24 h in the same spots for comparison.

### *In vitro* transwell (invasion and migration) assay

In vitro transwell (invasion) assay was performed by a modified method, briefly, 3 × 10^4^ cells in 150 μL serum-free medium supplemented with 1% FBS were seeded into the upper chamber of the insert (growth surface area, 0.33 cm^2^; membrane pore size, 8 μm; Corning Incorporated; Corning, NY, USA) with Matrigel (BD Biosciences, MA), and 500 μL medium supplemented with 10% FBS was added into the lower chamber of 24-well plastic plate. After 24 h of incubation at 37°C, the cells remained in the upper chamber or on the membrane were removed. Cells adhering to the lower membrane of the inserts were stained with DAPI after which were captured with confocal microscopy. The numbers of cells were counted in the images. Transwell (migration) assay was conducted the same as described above but not with Matrigel.

### Statistical analysis

Each experiment was done at least twice and at least one duplicate. The results were presented as mean ± SD. All calculations including statistical analysis were done by one-way ANOVA (SPSS 18.0). miRNA target prediction and associated mRNA pathway analysis were done using Ingenuity Pathway Analysis and TargetScan. Differences between treatments were assessed using Fisher’s Least Significant Difference test (LSD (L)). Significant difference was inferred for *P* < 0.05 and extremely significant difference *P* < 0.01 and *P* < 0.001.

## Results

### miR-200c expression is decreased in bladder cancer tissues and cell lines

**Figure 1:**
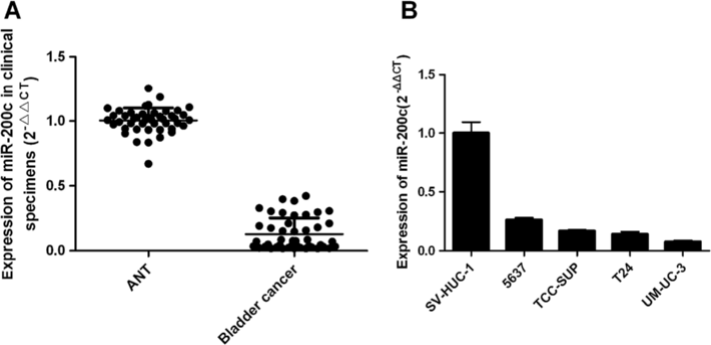
**miR-200c was down-regulated in bladder cancer tissues and cell lines. A**: Relative expression of miR-200c in 15 pairs of Bladder Cancer tissues and their corresponding adjacent noncancerous tissues (ANT). **B**: Different expressions of miR-200c in immortalized human nephric tubule cell line SV-HUC-1 and four bladder cancer cell lines (5637, TCC-SUP, T24, UMUC-3).

To investigate the potential significance of miR-200c in the development and progression of bladder cancer, we firstly examined the expression of the miR-200c in bladder cancer cell lines and clinical specimens, and found that miR-200c was ubiquitously expressed at lower levels in a panel of 4 human bladder cancer cell lines than in cultured immortalized human nephric tubule cell line SV-HUC-1 (Figure 1B). In parallel, as shown in Figure 1A, miR-200c expression was found to be markedly decreased in all 15 collected bladder cancer lesions as compared with that in paired adjacent non-cancerous bladder tissues. These data suggested that miR-200c expression was significantly suppressed in bladder cancer.

### miR-200c suppressed cell invasion, migration and proliferation in bladder cancer cells

**Figure 2:**
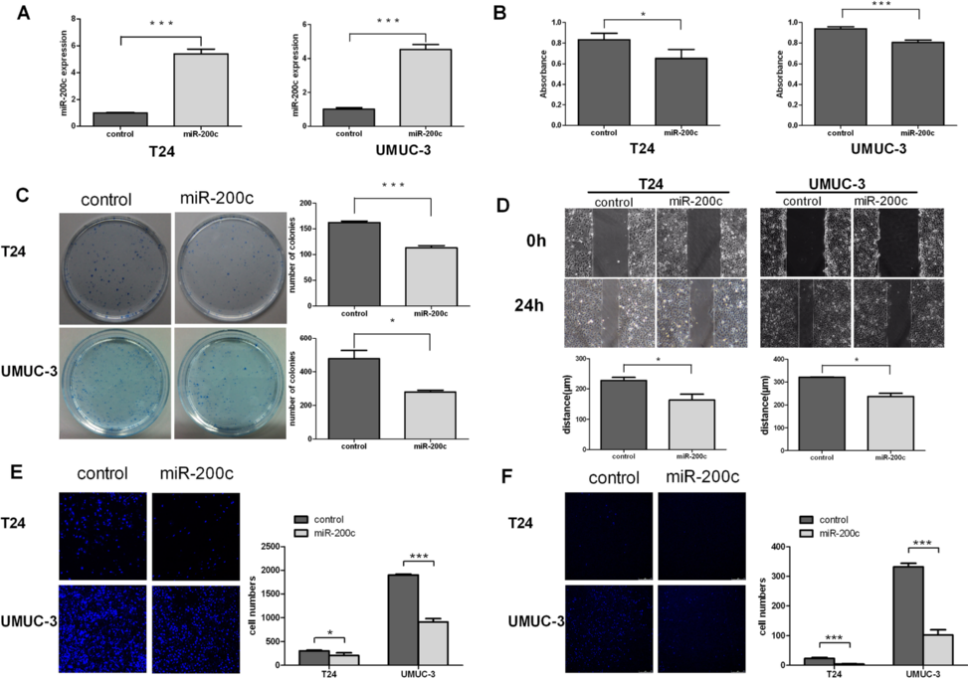
**Up-regulated miR-200c inhibited proliferation, migration and invasion in bladder cancer cells. A**: Entivirus carrying miR-200c plasmid and the control plasmid were persistently co-transfected into UMUC-3 and T24 cells. Measurements by real-time RT-PCR of miR-200c confirmed our success of transduction and were obviously higher than the control group in both cell lines. **B**: CCK-8 assays revealed cell growth differences of indicated cell lines. **C**: Colony formation assay in T24 and UMUC-3 cells. **D**: Measurement of *in vitro* cell migration by “wound-healing” assay. Representative pictures (left) and quantification (right) for same single spot in indicated cell lines. **E**: Transwell migration assays in indicated engineered cell lines. **F**: Transwell invasion assays in indicated engineered cell lines. Data are presented as mean ± SD from 3 independent experiments. *P < 0.05; **P < 0.01. ***P < 0.001; DAPI, 4’, 6-diamidino-2-phenylindole.

In the attempt to understand the biologic function of miR-200c, miR-200c was stably transduced into T24 and UMUC-3 cells through entivirus carrying miR-200c, respectively, to generate T24-miR-200c and UMUC-3-miR-200c cell lines. RT-PCR was used to confirm the success of transduction (Figure 2A). Figure 2H showed that over-expressed miR-200c inhibited invasion of T24 and UMUC-3 cells using a transwell essay, and decreased migration of T24 and UMUC-3 cells using wound-healing assay and transwell assay in miR-200c over-expression cells compared with control groups (Figure 2E, 2G). As shown in Figure 2B and 2C, ectopic miR-200c expression slowed down the proliferation of the bladder cancer cells, as analyzed with CCK-8 assay. Consisted with cell proliferation results, the capacities of colony formation of both cells were robustly compromised by miR-200c transduction as compared with corresponding control cells (Figure 2D and 2F). Taken together, these results suggested that over-expressed miR-200c suppressed the ability of bladder cancer cells to invade, migrate and proliferate *in vitro*.

### Antagonizing miR-200c accelerated cell invasion, migration and proliferation in bladder cancer cells

**Figure 3:**
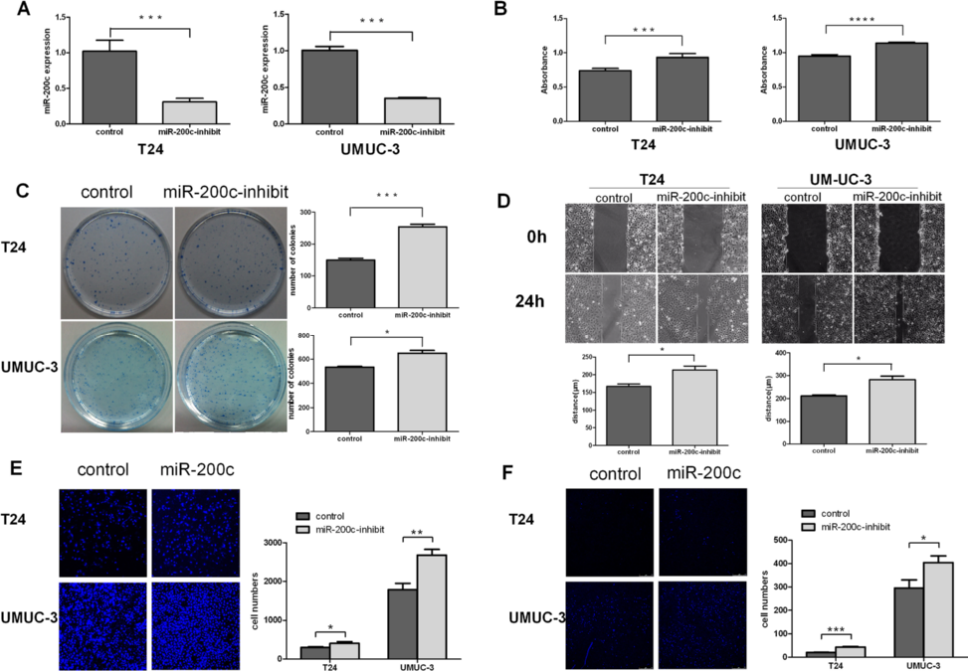
**Down-regulated miR-200c promoted growth and metastasis in bladder cancer cells. A**: Antagonizing endogenous miR-200c in UMUC-3 and T24 cells. Level of miR-200c was measured by real-time PCR and miR-200c was obviously antagonized in the both cell lines. **B**: CCK-8 assays revealed cell proliferation differences of T24 and UMUC-3 cells. **C**: Colony formation assay in indicated cell lines. **D**: Measurement of *in vitro* cell migration by wound-healing assay. **E**: Transwell migration assays in T24 and UMUC-3 cells. **F**: Transwell invasion cells in T24 and UMUC-3 cells. Data are presented as mean ± SD from 3 independent experiments. *P < 0.05; **P < 0.01. ***P < 0.001; DAPI, 4’, 6-diamidino-2-phenylindole.

To understand the role of endogenous miR-200c in the modulation of cell invasion, migration and proliferation, anti-miR-200c oligonucleotides were synthesized and used as antagonists to silence endogenous miR-200c expression (Figure 3A). As shown in Figure 3H and 3G, antagonized miR-200c drastically accelerated invasion, migration exhibited with transwell essay as compared with their corresponding vector-control cells in T24 and UMUC-3 cells, and the wound-healing assay showed the same tendency. In parallel, the ability of proliferation was also increased as showed in Figure 3B, C, D and F, analyzed using CCK-8 assay and colonies formation assay.

### miR-200c down-regulated BMI-1 and increases E-cadherin expression levels in bladder cancer cells

**Figure 4:**
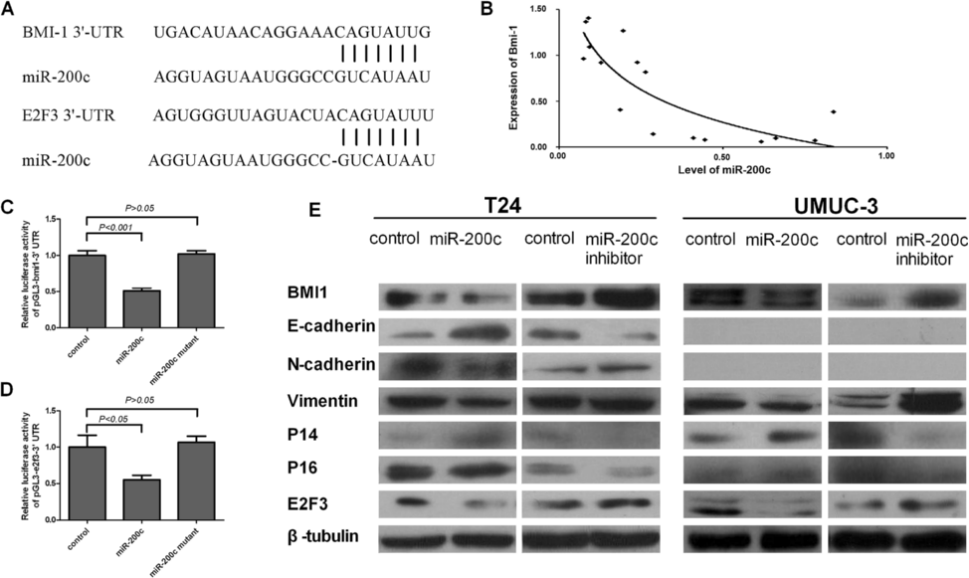
**miR-200c regulated the growth and metastasis of bladder cancer by directly targeting to BMI-1 and E2F3. A**: miR-200c directly targeted BMI-1. miR-200c directly targeted E2F3. **B**: Relationship between BMI-1 and miR-200c in the mRNA level. 16 tissue specimens were tested to measure the relationship between BMI-1 mRNA and miR-200c level, and found inverse correlation between BMI-1 and miR-200c. **C** and **D**: luciferase activity of pGL3-BMI-1-3’-UTR, pGL3-E2F3-3’-UTR reporter in indicated cells cotransfected with indicated oligonucleotides. **E**: Western blotting of BMI-1, E-cadherin, N-cadherin, Vimentin, P14, P16, and E2F3 expression in indicated cells. β-tubulin was used as a loading control.

By using TargetScan, a bioinformatic tool for miRNA target screening, we found that oncogene BMI-1 were tentative targets of miR-200c (Figure 4A).We also clarified it with luciferase assay that transfection of miR-200c mimic oligonucleotides abrogated the expression of luciferase (Figure 4C). However, mutating nucleotides in the seed sequence of miR-200c mimic oligonucleotides completely abolished their binding to the target 3’-UTRs. Indeed, the expression of BMI-1 inversely correlated with miR-200c expression in 16 tissue specimens (Figure 4B) at mRNA levels. Together, these results demonstrate a progressive diminution of miR-200c expression in bladder cancer compared with coordinated normal bladder tissues and suggest a further reduction in expression during bladder cancer progression, and BMI-1 expression correlates inversely with miR-200c expression.

To understand the mechanism underlying the robust suppressive effect of miR-200c on the invasion and migration of bladder cancer cells, more mechanism related research had been done. We asked whether enforced expression of miR-200c would also affect BMI-1 expression in bladder cancer cells. As shown in Figure 4E, in both T24 and UMUC-3 cell lines, western blotting confirmed that the protein levels of BMI-1 was indeed reduced drastically in miR-200c–transduced cells but pronouncedly elevated in miR-200c–silenced cells, as compared with those in the corresponding control cells. In parallel, miR-200c had great impact on the mRNA levels of these putative targets (Additional file 1: Figure S1). In consistence with these results, E-cadherin increased in miR-200c over-expressed cell lines but was down-regulated in miR-200c–inhibited cells of T24 (Figure 4E). Over-expressed miR-200c down-regulated production of mesenchymal marker N-cadherin and vimentin, but up-regulated E-cadherin expression in T24 cells (Figure 4E). BMI-1 has been proved to suppress the transcription of p16 and p14 in many tumors [16,17,40]. We wonder that how will p16 and p14 change after miRNA transfected. As Figure 4E showed, P16 and P14 were up-regulated in miR-200c over-expressed cells of T24 and UMUC-3 cells but the opposite in the miR-200c antagonized cells.

### miR-200c down-regulated E2F3 in bladder cancer cell lines

To determine the molecular mechanism of inducing cell growth arrest related with E2F3, we used 3 open-target prediction programs (i.e., picTar, TargetScan, and miRnada) to predict the targets of miR-200c. E2F3 was one of the oncogenic genes that were potential miR-200c targets. The 3’-UTR of E2F3 mRNA contained a complementary site for the seed region of miR-200c (Figure 4A). To determine whether E2F3 is the direct target gene for miR-200c, a dual-luciferase reporter system was employed. The luciferase reporter assay indicated that the luciferase activity of the reporter containing the E2F3 gene’s wild-type 3’-UTR decreased (52%) following treatment with miR-200c mimics. qRT-PCR was applied to determine how the mRNA levels of E2F3 changed in response to miR-200c transfection (Additional file 1: Figure S1). Results revealed that the expression of E2F3 mRNA and protein was inhibited by treatment with miR-200c transfected both in T24 and UMUC-3 cells (Figure 4E). By contrast, the inhibitory effect of the MiR-200c mimics was abolished in the mutated construct (Figure 4D). Taken together these data suggest that miR-200c reduces E2F3 expression by inhibiting translation and/or causing mRNA instability.

## Discussion

The current study has indicated that miR-200c is a tumor-suppressive miRNA in bladder cancer, and that oncogene and transcription promoters, namely, BMI-1 and E2F3, are functional targets of miR-200c. Our data demonstrated that miR-200c is remarkably down-regulated in bladder cancer cell lines and surgically excised bladder tumors. In this context, we have found that experimental up-regulation of miR-200c expression in bladder cancer cells leads to suppression of BMI-1 and E2F3, and disrupted invasion, migration and proliferation of the bladder cancer cells, whereas completely silencing miR-200c further up-regulates BMI-1 and E2F3 and promotes progression. Furthermore, inverse correlation between miR-200c level and expression of BMI-1, as well as tumor progression, is evidenced in our clinical relevance study. Particularly, miR-200c over-expression is significantly associated with improved survival in patients with bladder cancer, further suggesting a tumor-suppressive function of the molecule.

Interestingly, miR-200c is up-regulated in pancreatic cancer and the ability of proliferation of which was strengthen [42]. Moreover, a study by LIU [43] and colleagues suggests that the expression levels of miR-200c in non-small-cell lung cancer [NSCLC] were higher than those in normal tissues, and high expressions of tumor miR-200c was associated with a poor survival in NSCLC patients. On the other hand, however, Chang [18] and colleagues found that miR-200c inhibits metastasis of breast cancer cells by targeting HMGB1.

But how could miR-200c mediated the tumor suppressive effects? By miRNA target prediction and dual reporter gene, we confirmed oncogene BMI-1 as a directly target of miR-200c. And up-regulation of miR-200c decreased the BMI-1 gene expression, which inhibited the process of bladder cancer [44]. Chang and Boominathan L [45,46] and colleagues reported that miR-200c was a direct target of p53 transcription factor. P53 could inhibit cell proliferation through cell cycle arrest [29,30]. In this study we also found that over-expression of miR-200c caused similar effects that inhibited proliferation. It was also reported that over-expression of BMI-1 could cause p53 suppression, which provided evidence for the existence of some positive feedbacks [47,48]. In our study, we up-regulated BMI-1 through down-expression of miR-200c and found the approximate phenomenon, increased proliferation. BMI-1 expression inversely correlates with expressions of p14 and p16 in colorectal carcinomas [31]. BMI-1 has also been reported as an oncogene by regulating cell cycle inhibitors, p16 and p14 [49], which our consequences also proved. p16 was identified as an inhibitor of cyclin-dependent kinase 4 and 6 and thereby leads to hypophosphorylation of pRb [49]. On the other hand, p14ARF prevents the degradation and inactivation of p53 by binding to MDM-2 [50]. BMI-1, as a molecule of accelerating tumor progress, will promote proliferation of bladder cancer cells. Our results clearly demonstrate that ectopic miR-200c inhibits cell proliferation in T24 and UMUC-3 bladder cancer cells. Speaking of inhibition of cell proliferation, E2F3 maybe one of the targets miR-200c modulated that played a role in it. E2F3 is a member of the E2F family that is often dysregulated during tumorigenesis and over-expressed in a variety of cancers, including bladder cancer [39]. Recent studies suggest a critical role of E2F3 in gene expression control during the G1/S transition. Inhibitory antibodies against E2F3 blocked cell entry into the S phase [51]. In our study, we found that E2F3 was down-regulated in the miR-200c over-expression bladder cancer cell lines and consequently the proliferation was inhibited which was proved by CCK-8 assay and clone formation assay.

Several recent publications have suggested that regulation of the epithelial phenotype contributes to the miR-200c by acting on the E-cadherin repressors such as ZEB1/2 [17,52], the predominant target and negative regulators of miR-200c, through which the capabilities of invasion and migration of cancer cells was regulated. In our study, we found the same tendency; miR-200c directly mediated transcriptional up-regulation of E-cadherin through translational repression of ZEB1, the same as in MDAMB-231 cells [53], and down-regulation of the mesenchymal marker N-cadherin and vimentin. We also obtained the results of invasion and migration which were coincident with EMT hallmark E-cadherin.

By conducting the current study, we provide compelling biologic as well as clinical evidence that in bladder cancer miR-200c expression is markedly down-regulated, and that miR-200c acts to suppress the invasion, migration and proliferation of bladder cancer cells *in vitro*, suggesting that miR-200c plays a tumor suppressive role in the cancer type. These findings together indicate a dual role of miR-200c as either a tumor-promoting or suppressive miRNA, underscoring the need to define the specific role of a miRNA in a certain type of cancer. In this context, genetic and micro-environmental cues are believed to be important to determining whether, and how, a miRNA molecule functions to promote or to suppress oncogenes. Thus, it remains important to thoroughly understand the molecular mechanisms mediating the differential biologic effects and targets of miR-200c in bladder cancer and other cancer types.

Our current study has identified invasion, migration and proliferation regulator BMI-1, proliferation regulator E2F3 which was a transcriptional factor as bonafide targets of miR-200c. These genes control the characters of invasion, migration and proliferation and thus BMI-1 have been implicated as proto-oncogenes and E2F3 the transcriptional factor attractive therapeutic targets against cancer. Over-expression of BMI-1 is one of the hallmarks of various cancer types, due to its relatively high amplification frequency and up-regulated expression of its mRNA and protein in a variety of tumors including bladder cancer, kidney cancer, and breast cancer.

The research above, on the one hand provide new insights in our need to develop more efficacious therapeutic approach to improved treatment of bladder cancer. On the other hand, however, the mechanism via which miR-200c is down-regulated in bladder cancer remains uninvestigated.

## Conclusions

Taken together, it would be of great interest to further investigate whether miR-200c down-regulation in bladder cancer was attributable to genomic deletion and/or promoter methylation. In summary, on the backdrop that bladder cancer remains a highly challenging and deadly malignancy, in addition to searching for novel therapeutic agents, it is equally important to identify effective biomarkers for diagnosis and prognosis of bladder cancer. To this end, our present finding that the level of miR-200c expression correlates with the clinic pathologic features and patient survival in bladder cancer makes it a reasonable candidate biomarker for determination of the prognosis of the disease. Thus, these findings warrant further investigation on the potential development of miR-200c-based prognostic and therapeutic approaches.

## Additional file

Additional file 1: Figure S1. Expression of relative mRNAs in T24 and UMUC-3 cells. miR-200c was up-regulated or down-regulated in T24 and UMUC-3 cells, and expression of BMI-1 (A), E2F3 (B), E-cadherin (C) and Vimentin (D) were measured by real-time PCR. Data are presented as mean ± SD from 3 independent experiments. **P* < 0.05; ***P* < 0.01. ****P* < 0.001; DAPI, 4’, 6-diamidino-2-phenylindole.
