# Correction to: miR-200c Inhibits invasion, migration and proliferation of bladder cancer cells through down-regulation of BMI-1 and E2F3

**DOI:** 10.1186/s12967-022-03299-6

**Published:** 2022-04-05

**Authors:** Lei Liu, Mingning Qiu, Guobin Tan, Ziji Liang, Yue Qin, Lieqian Chen, Hege Chen, Jianjun Liu

**Affiliations:** Laboratory of Urology, Guangdong Medical College, Zhanjiang, 524001 China

## Correction to: Journal of Translational Medicine (2014) 12:305 https://doi.org/10.1186/s12967-014-0305-z

Following publication of the original article [1], we have been notified by the authors that Figures in the article should be revised. Correct figures are mentioned in this article (Figs. 1, 2 and 3).

**Fig. 1:**
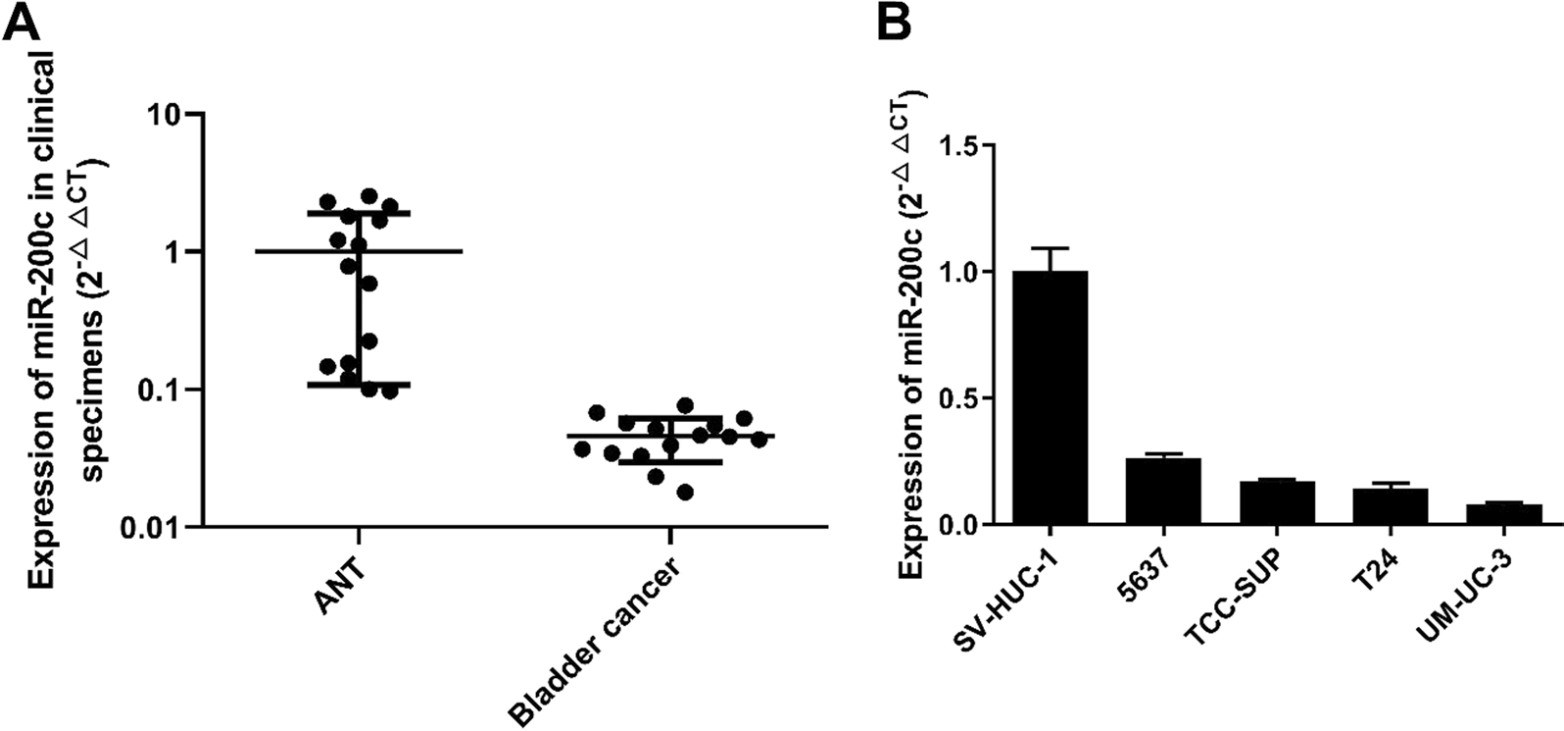
miR-200c was down-regulated in bladder cancer tissues and cell lines. **A** Relative expression of miR-200c in 15 pairs of Bladder Cancer tissues and their corresponding adjacent noncancerous tissues (ANT). **B** Different expressions of miR-200c in immortalized human nephric tubule cell line SV-HUC-1 and four bladder cancer cell lines (5637, TCC-SUP, T24, UMUC-3)

**Fig. 2:**
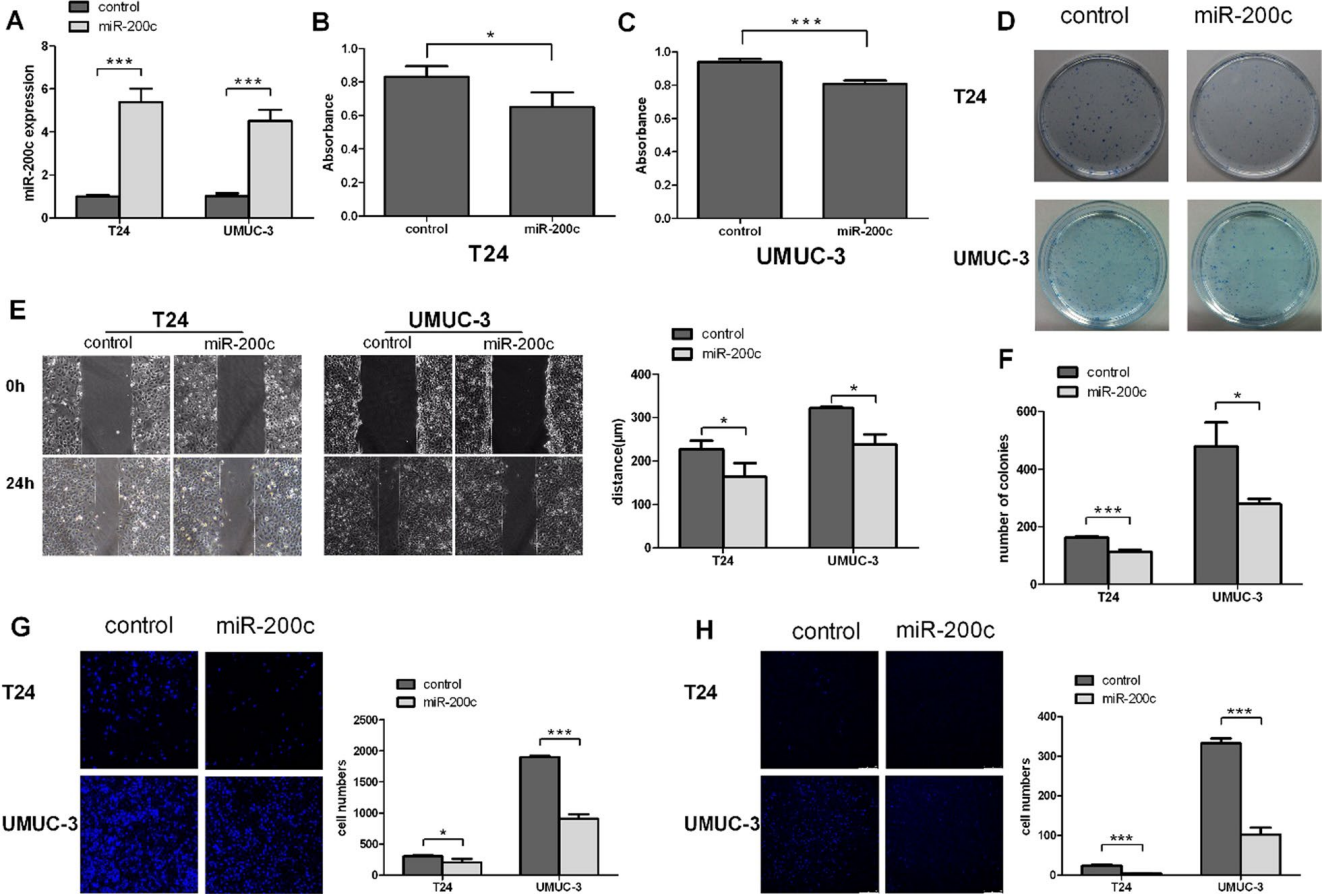
Up-regulated miR-200c inhibited proliferation, migration and invasion in bladder cancer cells. **A** Entivirus carrying miR-200c plasmid and the control plasmid were persistently co-transfected into UMUC-3 and T24 cells. Measurements by real-time RT-PCR of miR-200c confirmed our success of transduction and were obviously higher than the control group in both cell lines. **B** CCK-8 assays revealed cell growth differences of indicated cell lines. **C** Colony formation assay in T24 and UMUC-3 cells. **D** Measurement of in vitro cell migration by “wound-healing” assay. Representative pictures (left) and quantification (right) for same single spot in indicated cell lines. **E** Transwell migration assays in indicated engineered cell lines. **F** Transwell invasion assays in indicated engineered cell lines. Data are presented as mean ± SD from 3 independent experiments. *P < 0.05; **P < 0.01. ***P < 0.001; DAPI, 4′,6-diamidino-2-phenylindole

**Fig. 3:**
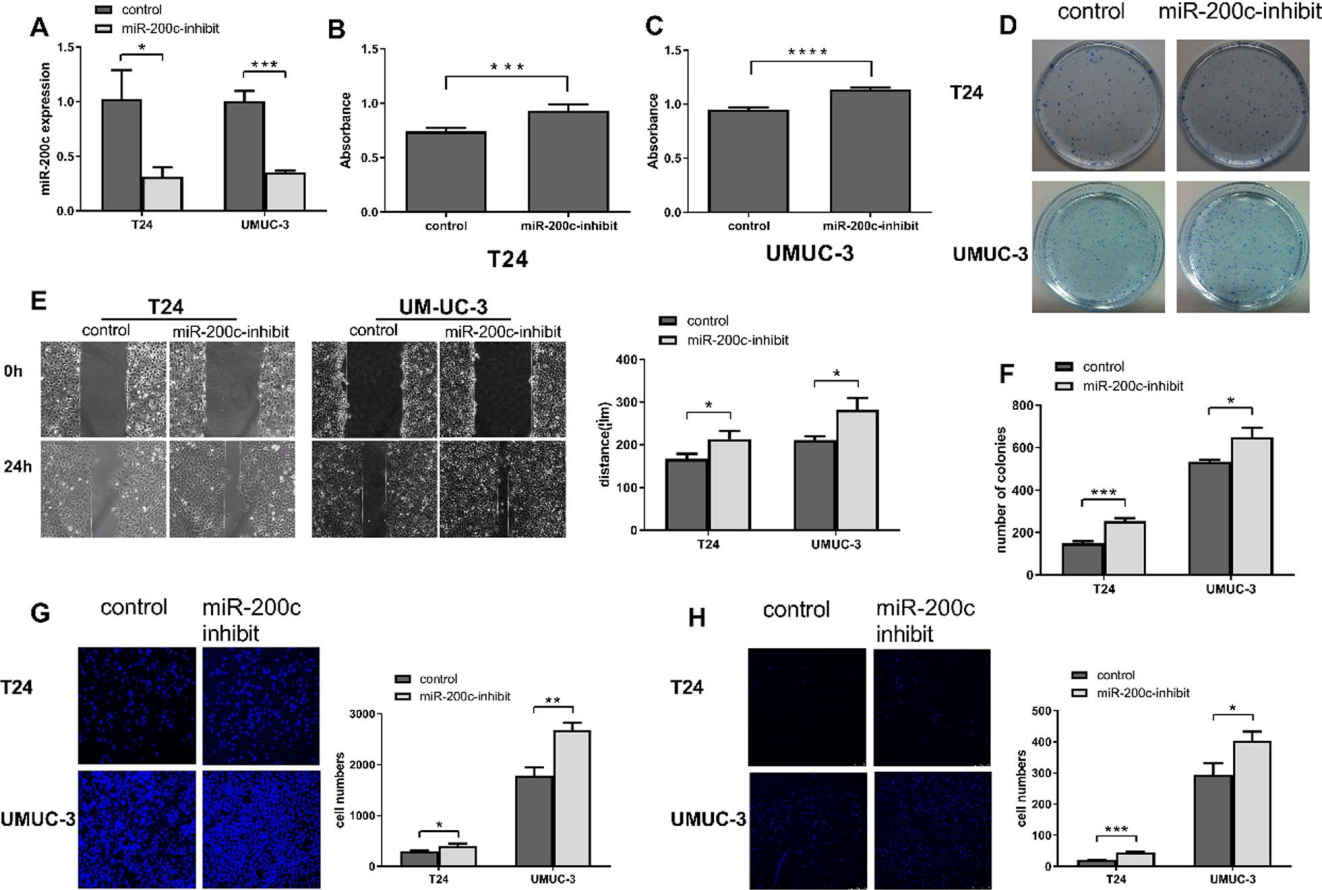
Down-regulated miR-200c promoted growth and metastasis in bladder cancer cells. **A** Antagonizing endogenous miR-200c in UMUC-3 and T24 cells. Level of miR-200c was measured by real-time PCR and miR-200c was obviously antagonized in the both cell lines. **B** CCK-8 assays revealed cell proliferation differences of T24 and UMUC-3 cells. **C** Colony formation assay in indicated cell lines. **D** Measurement of in vitro cell migration by wound-healing assay. **E** Transwell migration assays in T24 and UMUC-3 cells. **F** Transwell invasion cells in T24 and UMUC-3 cells. Data are presented as mean ± SD from 3 independent experiments. *P < 0.05; **P < 0.01. ***P < 0.001; DAPI, 4′,6-diamidino-2-phenylindole
