# Supplementary figures and images for: miR-200c Inhibits invasion, migration and proliferation of bladder cancer cells through down-regulation of BMI-1 and E2F3
Source: J Transl Med. 2014 Nov 4;12:305. doi: 10.1186/s12967-014-0305-z (PMC4226852; doi:10.1186/s12967-014-0305-z)

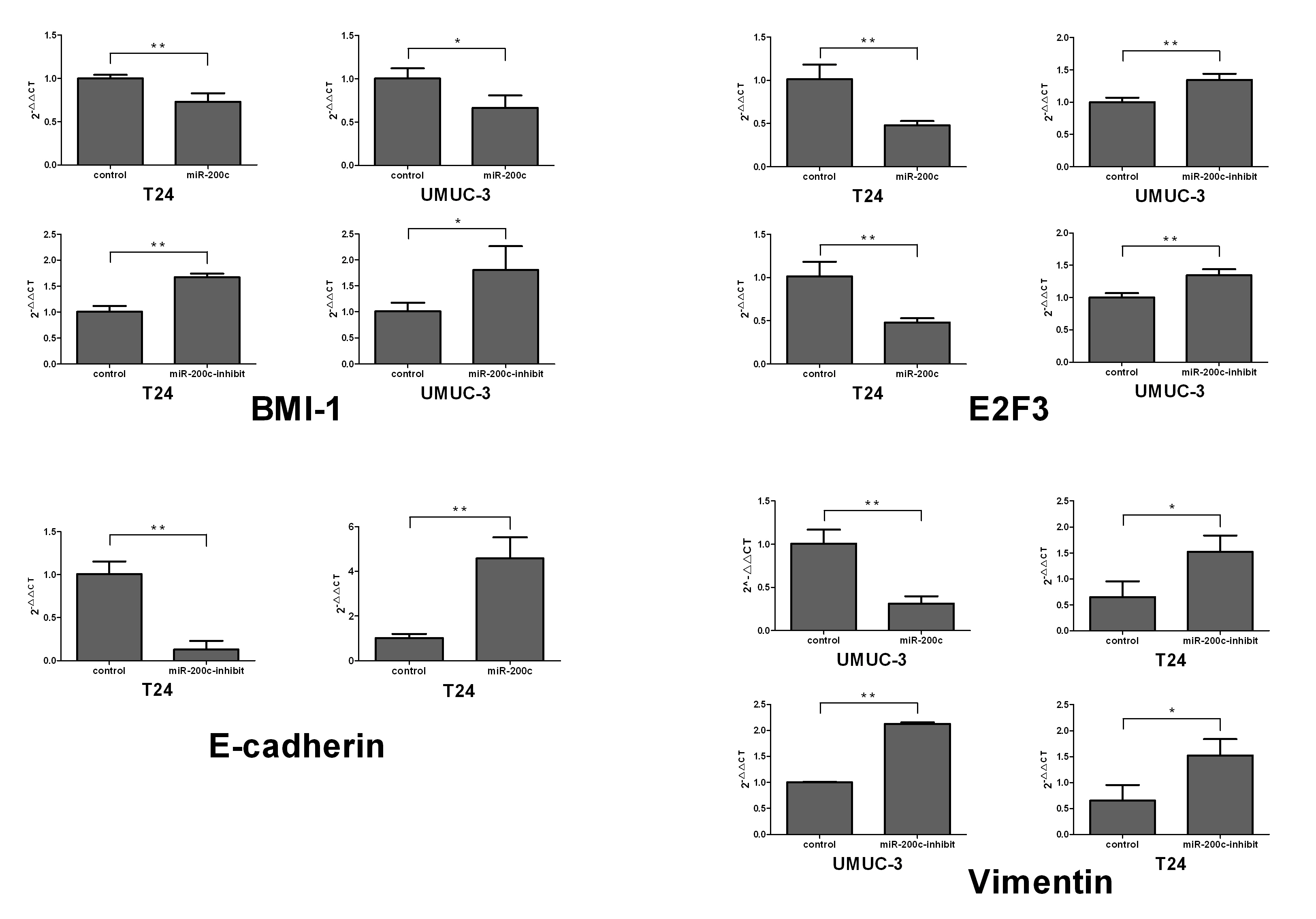

Supplement: Additional file 1: Figure S1. — Expression of relative mRNAs in T24 and UMUC-3 cells. miR-200c was up-regulated or down-regulated in T24 and UMUC-3 cells, and expression of BMI-1 (A), E2F3 (B), E-cadherin (C) and Vimentin (D) were measured by real-time PCR. Data are presented as mean ± SD from 3 independent experiments. *P < 0.05; **P < 0.01. ***P < 0.001; DAPI, 4’, 6-diamidino-2-phenylindole. [file 12967_2014_305_MOESM1_ESM.jpeg]
